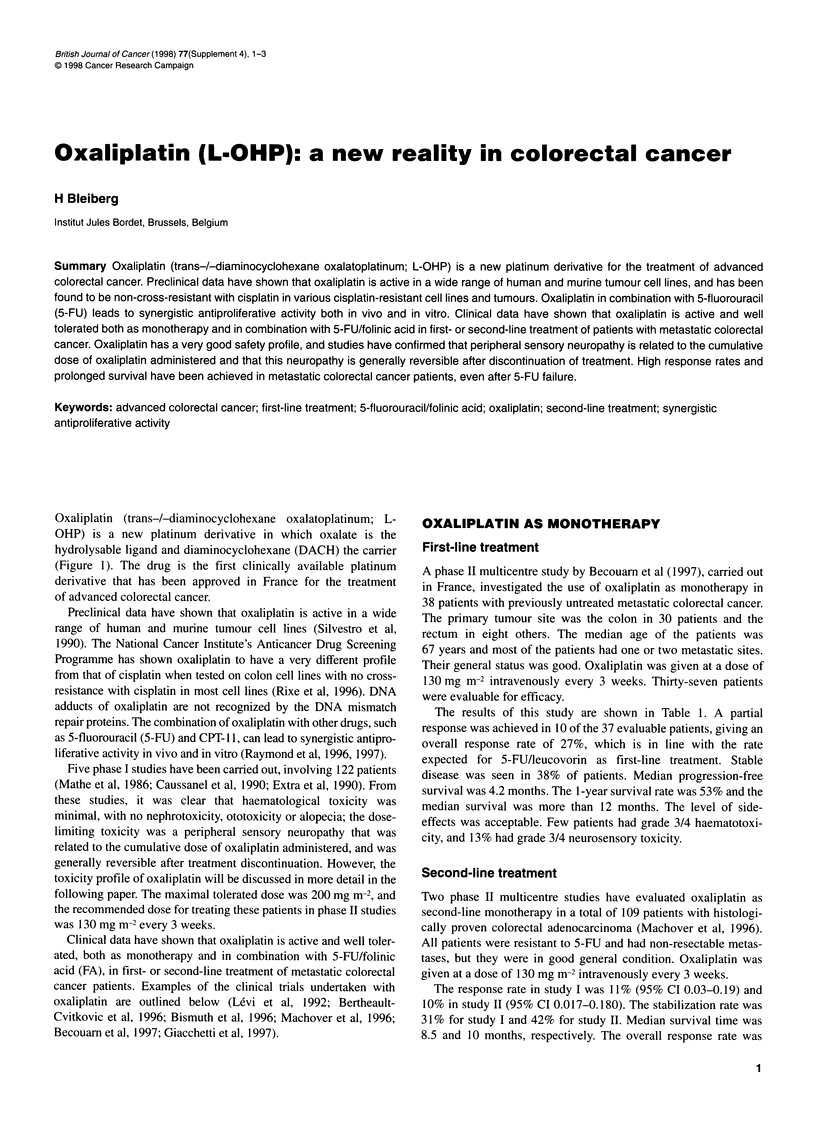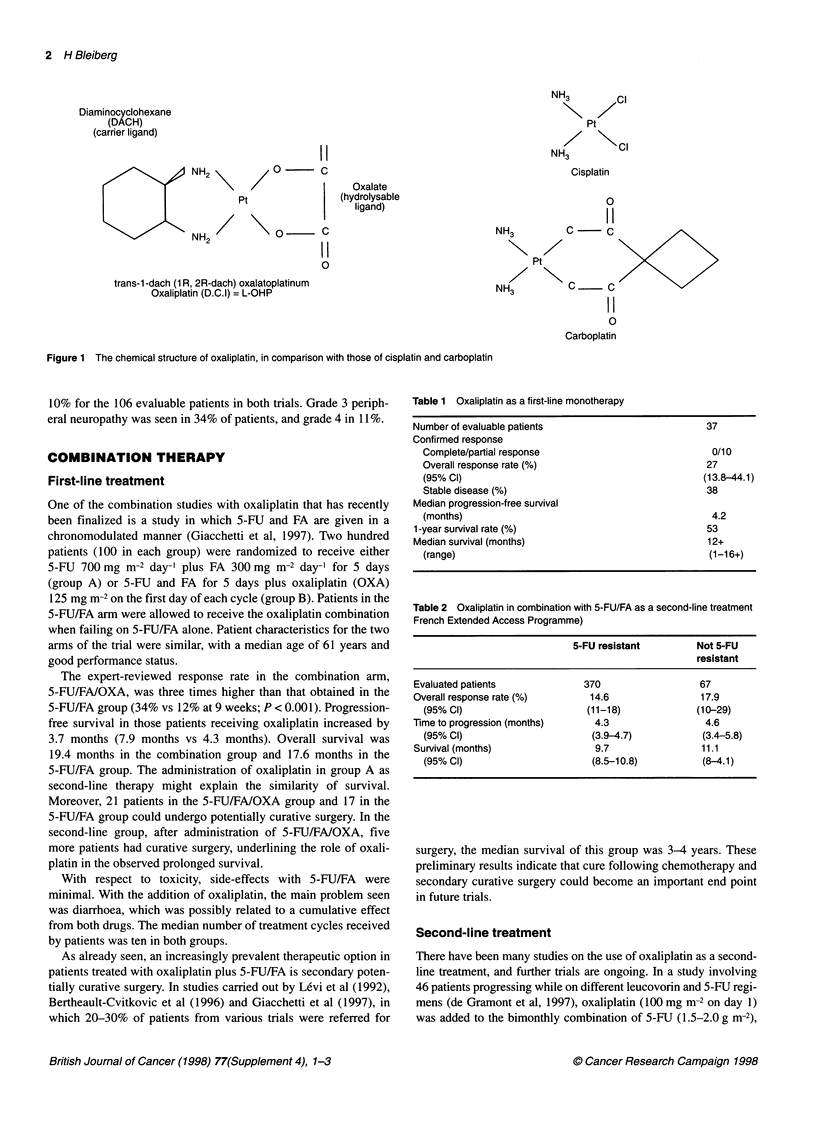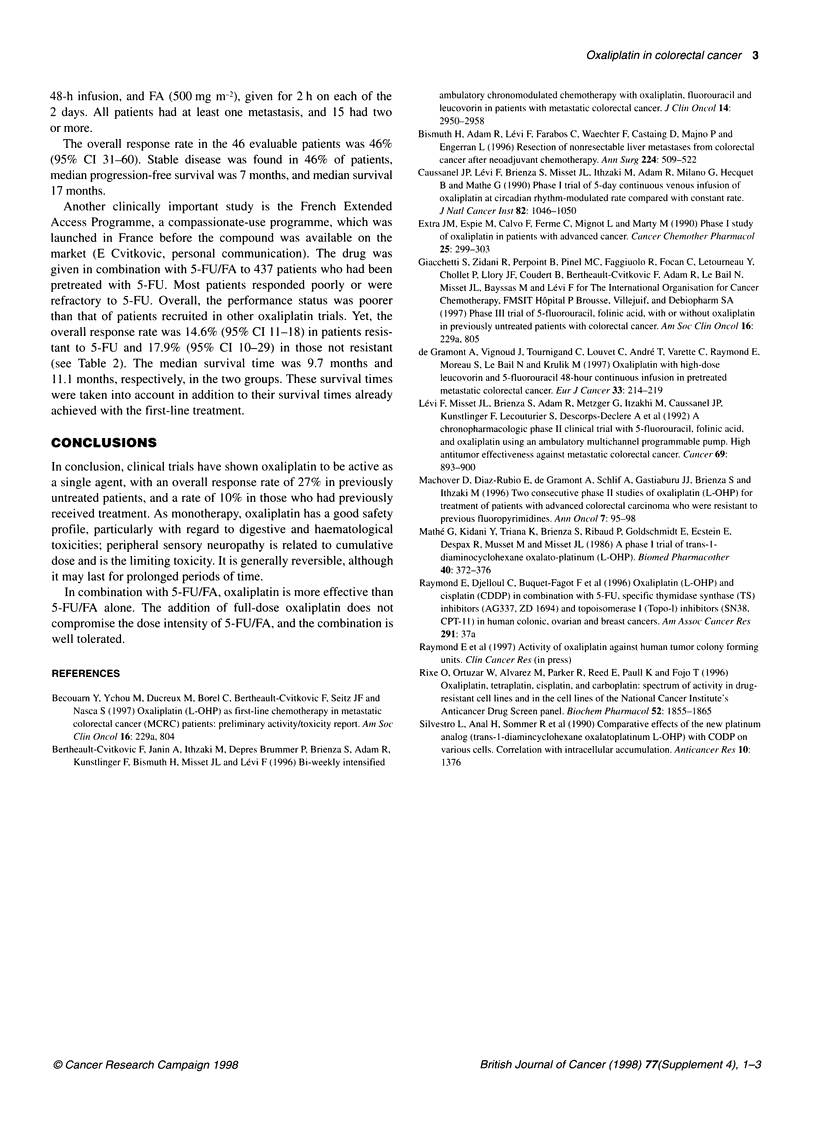# Oxaliplatin (L-OHP): a new reality in colorectal cancer.

**DOI:** 10.1038/bjc.1998.427

**Published:** 1998-06

**Authors:** H. Bleiberg

**Affiliations:** Institut Jules Bordet, Brussels, Belgium.

## Abstract

Oxaliplatin (trans-/-diaminocyclohexane oxalatoplatinum; L-OHP) is a new platinum derivative for the treatment of advanced colorectal cancer. Preclinical data have shown that oxaliplatin is active in a wide range of human and murine tumour cell lines, and has been found to be non-cross-resistant with cisplatin in various cisplatin-resistant cell lines and tumours. Oxaliplatin in combination with 5-fluorouracil (5-FU) leads to synergistic antiproliferative activity both in vivo and in vitro. Clinical data have shown that oxaliplatin is active and well tolerated both as monotherapy and in combination with 5-FU/folinic acid in first- or second-line treatment of patients with metastatic colorectal cancer. Oxaliplatin has a very good safety profile, and studies have confirmed that peripheral sensory neuropathy is related to the cumulative dose of oxaliplatin administered and that this neuropathy is generally reversible after discontinuation of treatment. High response rates and prolonged survival have been achieved in metastatic colorectal cancer patients, even after 5-FU failure.


British Journal of Cancer (1998) 77(Supplement 4), 1–3
© 1998 Cancer Research Campaign

### Oxaliplatin (L-OHP): a new reality in colorectal cancer

**H Bleiberg**

Institut Jules Bordet, Brussels, Belgium

**Summary** Oxaliplatin (trans-/-diaminocyclohexane oxalatoplatinum; L-OHP) is a new platinum derivative for the treatment of advanced colorectal cancer. Preclinical data have shown that oxaliplatin is active in a wide range of human and murine tumour cell lines, and has been found to be non-cross-resistant with cisplatin in various cisplatin-resistant cell lines and tumours. Oxaliplatin in combination with 5-fluorouracil (5-FU) leads to synergistic antiproliferative activity both in vivo and in vitro. Clinical data have shown that oxaliplatin is active and well tolerated both as monotherapy and in combination with 5-FU/folinic acid in first- or second-line treatment of patients with metastatic colorectal cancer. Oxaliplatin has a very good safety profile, and studies have confirmed that peripheral sensory neuropathy is related to the cumulative dose of oxaliplatin administered and that this neuropathy is generally reversible after discontinuation of treatment. High response rates and prolonged survival have been achieved in metastatic colorectal cancer patients, even after 5-FU failure.

**Keywords:** advanced colorectal cancer; first-line treatment; 5-fluorouracil/folinic acid; oxaliplatin; second-line treatment; synergistic antiproliferative activity

Oxaliplatin (trans-/-diaminocyclohexane oxalatoplatinum; L-OHP) is a new platinum derivative in which oxalate is the hydrolysable ligand and diaminocyclohexane (DACH) the carrier (Figure 1). The drug is the first clinically available platinum derivative that has been approved in France for the treatment of advanced colorectal cancer.

Preclinical data have shown that oxaliplatin is active in a wide range of human and murine tumour cell lines (Silvestro et al, 1990). The National Cancer Institute's Anticancer Drug Screening Programme has shown oxaliplatin to have a very different profile from that of cisplatin when tested on colon cell lines with no cross-resistance with cisplatin in most cell lines (Rixe et al, 1996). DNA adducts of oxaliplatin are not recognized by the DNA mismatch repair proteins. The combination of oxaliplatin with other drugs, such as 5-fluorouracil (5-FU) and CPT-11, can lead to synergistic antiproliferative activity in vivo and in vitro (Raymond et al, 1996, 1997).

Five phase I studies have been carried out, involving 122 patients (Mathe et al, 1986; Caussanel et al, 1990; Extra et al, 1990). From these studies, it was clear that haematological toxicity was minimal, with no nephrotoxicity, ototoxicity or alopecia; the dose-limiting toxicity was a peripheral sensory neuropathy that was related to the cumulative dose of oxaliplatin administered, and was generally reversible after treatment discontinuation. However, the toxicity profile of oxaliplatin will be discussed in more detail in the following paper. The maximal tolerated dose was 200 mg $m^{-2}$, and the recommended dose for treating these patients in phase II studies was 130 mg $m^{-2}$ every 3 weeks.

Clinical data have shown that oxaliplatin is active and well tolerated, both as monotherapy and in combination with 5-FU/folinic acid (FA), in first- or second-line treatment of metastatic colorectal cancer patients. Examples of the clinical trials undertaken with oxaliplatin are outlined below (Lévi et al, 1992; Bertheault-Cvitkovic et al, 1996; Bismuth et al, 1996; Machover et al, 1996; Becouarn et al, 1997; Giacchetti et al, 1997).

### OXALIPLATIN AS MONOTHERAPY

### First-line treatment

A phase II multicentre study by Becouarn et al (1997), carried out in France, investigated the use of oxaliplatin as monotherapy in 38 patients with previously untreated metastatic colorectal cancer. The primary tumour site was the colon in 30 patients and the rectum in eight others. The median age of the patients was 67 years and most of the patients had one or two metastatic sites. Their general status was good. Oxaliplatin was given at a dose of 130 mg $m^{-2}$ intravenously every 3 weeks. Thirty-seven patients were evaluable for efficacy.

The results of this study are shown in Table 1. A partial response was achieved in 10 of the 37 evaluable patients, giving an overall response rate of 27%, which is in line with the rate expected for 5-FU/leucovorin as first-line treatment. Stable disease was seen in 38% of patients. Median progression-free survival was 4.2 months. The 1-year survival rate was 53% and the median survival was more than 12 months. The level of side-effects was acceptable. Few patients had grade 3/4 haematotoxicity, and 13% had grade 3/4 neurosensory toxicity.

### Second-line treatment

Two phase II multicentre studies have evaluated oxaliplatin as second-line monotherapy in a total of 109 patients with histologically proven colorectal adenocarcinoma (Machover et al, 1996). All patients were resistant to 5-FU and had non-resectable metastases, but they were in good general condition. Oxaliplatin was given at a dose of 130 mg $m^{-2}$ intravenously every 3 weeks.

The response rate in study I was 11% (95% CI 0.03–0.19) and 10% in study II (95% CI 0.017–0.180). The stabilization rate was 31% for study I and 42% for study II. Median survival time was 8.5 and 10 months, respectively. The overall response rate was

**Figure 1** The chemical structure of oxaliplatin, in comparison with those of cisplatin and carboplatin

10% for the 106 evaluable patients in both trials. Grade 3 peripheral neuropathy was seen in 34% of patients, and grade 4 in 11%.

### COMBINATION THERAPY

### First-line treatment

One of the combination studies with oxaliplatin that has recently been finalized is a study in which 5-FU and FA are given in a chronomodulated manner (Giacchetti et al, 1997). Two hundred patients (100 in each group) were randomized to receive either 5-FU 700 mg $m^{-2}$ $day^{-1}$ plus FA 300 mg $m^{-2}$ $day^{-1}$ for 5 days (group A) or 5-FU and FA for 5 days plus oxaliplatin (OXA) 125 mg $m^{-2}$ on the first day of each cycle (group B). Patients in the 5-FU/FA arm were allowed to receive the oxaliplatin combination when failing on 5-FU/FA alone. Patient characteristics for the two arms of the trial were similar, with a median age of 61 years and good performance status.

The expert-reviewed response rate in the combination arm, 5-FU/FA/OXA, was three times higher than that obtained in the 5-FU/FA group (34% vs 12% at 9 weeks; $P < 0.001$). Progression-free survival in those patients receiving oxaliplatin increased by 3.7 months (7.9 months vs 4.3 months). Overall survival was 19.4 months in the combination group and 17.6 months in the 5-FU/FA group. The administration of oxaliplatin in group A as second-line therapy might explain the similarity of survival. Moreover, 21 patients in the 5-FU/FA/OXA group and 17 in the 5-FU/FA group could undergo potentially curative surgery. In the second-line group, after administration of 5-FU/FA/OXA, five more patients had curative surgery, underlining the role of oxaliplatin in the observed prolonged survival.

With respect to toxicity, side-effects with 5-FU/FA were minimal. With the addition of oxaliplatin, the main problem seen was diarrhoea, which was possibly related to a cumulative effect from both drugs. The median number of treatment cycles received by patients was ten in both groups.

As already seen, an increasingly prevalent therapeutic option in patients treated with oxaliplatin plus 5-FU/FA is secondary potentially curative surgery. In studies carried out by Lévi et al (1992), Bertheault-Cvitkovic et al (1996) and Giacchetti et al (1997), in which 20–30% of patients from various trials were referred for surgery, the median survival of this group was 3–4 years. These preliminary results indicate that cure following chemotherapy and secondary curative surgery could become an important end point in future trials.

**Table 1** Oxaliplatin as a first-line monotherapy

| | |
|---|---|
| Number of evaluable patients | 37 |
| Confirmed response | |
| Complete/partial response | 0/10 |
| Overall response rate (%) | 27 |
| (95% CI) | (13.8–44.1) |
| Stable disease (%) | 38 |
| Median progression-free survival (months) | 4.2 |
| 1-year survival rate (%) | 53 |
| Median survival (months) | 12+ |
| (range) | (1–16+) |

**Table 2** Oxaliplatin in combination with 5-FU/FA as a second-line treatment French Extended Access Programme)

| | 5-FU resistant | Not 5-FU resistant |
|---|---|---|
| Evaluated patients | 370 | 67 |
| Overall response rate (%) | 14.6 | 17.9 |
| (95% CI) | (11–18) | (10–29) |
| Time to progression (months) | 4.3 | 4.6 |
| (95% CI) | (3.9–4.7) | (3.4–5.8) |
| Survival (months) | 9.7 | 11.1 |
| (95% CI) | (8.5–10.8) | (8–4.1) |

### Second-line treatment

There have been many studies on the use of oxaliplatin as a second-line treatment, and further trials are ongoing. In a study involving 46 patients progressing while on different leucovorin and 5-FU regimens (de Gramont et al, 1997), oxaliplatin (100 mg $m^{-2}$ on day 1) was added to the bimonthly combination of 5-FU (1.5–2.0 g $m^{-2}$),

© Cancer Research Campaign 1998

48-h infusion, and FA (500 mg $m^{-2}$), given for 2 h on each of the 2 days. All patients had at least one metastasis, and 15 had two or more.

The overall response rate in the 46 evaluable patients was 46% (95% CI 31–60). Stable disease was found in 46% of patients, median progression-free survival was 7 months, and median survival 17 months.

Another clinically important study is the French Extended Access Programme, a compassionate-use programme, which was launched in France before the compound was available on the market (E Cvitkovic, personal communication). The drug was given in combination with 5-FU/FA to 437 patients who had been pretreated with 5-FU. Most patients responded poorly or were refractory to 5-FU. Overall, the performance status was poorer than that of patients recruited in other oxaliplatin trials. Yet, the overall response rate was 14.6% (95% CI 11–18) in patients resistant to 5-FU and 17.9% (95% CI 10–29) in those not resistant (see Table 2). The median survival time was 9.7 months and 11.1 months, respectively, in the two groups. These survival times were taken into account in addition to their survival times already achieved with the first-line treatment.

### CONCLUSIONS

In conclusion, clinical trials have shown oxaliplatin to be active as a single agent, with an overall response rate of 27% in previously untreated patients, and a rate of 10% in those who had previously received treatment. As monotherapy, oxaliplatin has a good safety profile, particularly with regard to digestive and haematological toxicities; peripheral sensory neuropathy is related to cumulative dose and is the limiting toxicity. It is generally reversible, although it may last for prolonged periods of time.

In combination with 5-FU/FA, oxaliplatin is more effective than 5-FU/FA alone. The addition of full-dose oxaliplatin does not compromise the dose intensity of 5-FU/FA, and the combination is well tolerated.

### REFERENCES

Becouarn Y, Ychou M, Ducreux M, Borel C, Bertheault-Cvitkovic F, Seitz JF and Nasca S (1997) Oxaliplatin (L-OHP) as first-line chemotherapy in metastatic colorectal cancer (MCRC) patients: preliminary activity/toxicity report. *Am Soc Clin Oncol* **16**: 229a, 804

Bertheault-Cvitkovic F, Janin A, Ithzaki M, Depres Brummer P, Brienza S, Adam R, Kunstlinger F, Bismuth H, Misset JL and Lévi F (1996) Bi-weekly intensified ambulatory chronomodulated chemotherapy with oxaliplatin, fluorouracil and leucovorin in patients with metastatic colorectal cancer. *J Clin Oncol* **14**: 2950–2958

Bismuth H, Adam R, Lévi F, Farabos C, Waechter F, Castaing D, Majno P and Engerran L (1996) Resection of nonresectable liver metastases from colorectal cancer after neoadjuvant chemotherapy. *Ann Surg* **224**: 509–522

Caussanel JP, Lévi F, Brienza S, Misset JL, Ithzaki M, Adam R, Milano G, Hecquet B and Mathe G (1990) Phase I trial of 5-day continuous venous infusion of oxaliplatin at circadian rhythm-modulated rate compared with constant rate. *J Natl Cancer Inst* **82**: 1046–1050

Extra JM, Espie M, Calvo F, Ferme C, Mignot L and Marty M (1990) Phase I study of oxaliplatin in patients with advanced cancer. *Cancer Chemother Pharmacol* **25**: 299–303

Giacchetti S, Zidani R, Perpoint B, Pinel MC, Faggiuolo R, Focan C, Letourneau Y, Chollet P, Llory JF, Coudert B, Bertheault-Cvitkovic F, Adam R, Le Bail N, Misset JL, Bayssas M and Lévi F for The International Organisation for Cancer Chemotherapy, FMSIT Hôpital P Brousse, Villejuif, and Debiopharm SA (1997) Phase III trial of 5-fluorouracil, folinic acid, with or without oxaliplatin in previously untreated patients with colorectal cancer. *Am Soc Clin Oncol* **16**: 229a, 805

de Gramont A, Vignoud J, Tournigand C, Louvet C, André T, Varette C, Raymond E, Moreau S, Le Bail N and Krulik M (1997) Oxaliplatin with high-dose leucovorin and 5-fluorouracil 48-hour continuous infusion in pretreated metastatic colorectal cancer. *Eur J Cancer* **33**: 214–219

Lévi F, Misset JL, Brienza S, Adam R, Metzger G, Itzakhi M, Caussanel JP, Kunstlinger F, Lecouturier S, Descorps-Declere A et al (1992) A chronopharmacologic phase II clinical trial with 5-fluorouracil, folinic acid, and oxaliplatin using an ambulatory multichannel programmable pump. High antitumor effectiveness against metastatic colorectal cancer. *Cancer* **69**: 893–900

Machover D, Diaz-Rubio E, de Gramont A, Schlif A, Gastiaburu JJ, Brienza S and Ithzaki M (1996) Two consecutive phase II studies of oxaliplatin (L-OHP) for treatment of patients with advanced colorectal carcinoma who were resistant to previous fluoropyrimidines. *Ann Oncol* **7**: 95–98

Mathé G, Kidani Y, Triana K, Brienza S, Ribaud P, Goldschmidt E, Ecstein E, Despax R, Musset M and Misset JL (1986) A phase I trial of trans-1-diaminocyclohexane oxalato-platinum (L-OHP). *Biomed Pharmacother* **40**: 372–376

Raymond E, Djelloul C, Buquet-Fagot F et al (1996) Oxaliplatin (L-OHP) and cisplatin (CDDP) in combination with 5-FU, specific thymidase synthase (TS) inhibitors (AG337, ZD 1694) and topoisomerase I (Topo-l) inhibitors (SN38, CPT-11) in human colonic, ovarian and breast cancers. *Am Assoc Cancer Res* **291**: 37a

Raymond E et al (1997) Activity of oxaliplatin against human tumor colony forming units. *Clin Cancer Res* (in press)

Rixe O, Ortuzar W, Alvarez M, Parker R, Reed E, Paull K and Fojo T (1996) Oxaliplatin, tetraplatin, cisplatin, and carboplatin: spectrum of activity in drug-resistant cell lines and in the cell lines of the National Cancer Institute's Anticancer Drug Screen panel. *Biochem Pharmacol* **52**: 1855–1865

Silvestro L, Anal H, Sommer R et al (1990) Comparative effects of the new platinum analog (trans-1-diamincyclohexane oxalatoplatinum L-OHP) with CODP on various cells. Correlation with intracellular accumulation. *Anticancer Res* **10**: 1376

© Cancer Research Campaign 1998